# Automated Information Extraction on Treatment and Prognosis for Non–Small Cell Lung Cancer Radiotherapy Patients: Clinical Study

**DOI:** 10.2196/medinform.8662

**Published:** 2018-02-01

**Authors:** Shuai Zheng, Salma K Jabbour, Shannon E O'Reilly, James J Lu, Lihua Dong, Lijuan Ding, Ying Xiao, Ning Yue, Fusheng Wang, Wei Zou

**Affiliations:** ^1^ Department of Biomedical Informatics Emory University Atlanta, GA United States; ^2^ Department of Radiation Oncology Rutgers Cancer Institute of New Jersey New Brunswick, NJ United States; ^3^ Penn Medicine Department of Radiation Oncology University of Pennsylvania Philadelphia, PA United States; ^4^ Department of Mathematics and Computer Science Emory University Atlanta, GA United States; ^5^ Department of Radiation Oncology The First Hospital Changchun China; ^6^ Department of Biomedical Informatics Stony Brook University Stony Brook, NY United States; ^7^ Department of Computer Science Stony Brook University Stony Brook, NY United States

**Keywords:** information extraction, oncology, chemoradiation treatment, prognosis, non–small cell lung, information storage and retrieval, natural language processing

## Abstract

**Background:**

In outcome studies of oncology patients undergoing radiation, researchers extract valuable information from medical records generated before, during, and after radiotherapy visits, such as survival data, toxicities, and complications. Clinical studies rely heavily on these data to correlate the treatment regimen with the prognosis to develop evidence-based radiation therapy paradigms. These data are available mainly in forms of narrative texts or table formats with heterogeneous vocabularies. Manual extraction of the related information from these data can be time consuming and labor intensive, which is not ideal for large studies.

**Objective:**

The objective of this study was to adapt the interactive information extraction platform Information and Data Extraction using Adaptive Learning (IDEAL-X) to extract treatment and prognosis data for patients with locally advanced or inoperable non–small cell lung cancer (NSCLC).

**Methods:**

We transformed patient treatment and prognosis documents into normalized structured forms using the IDEAL-X system for easy data navigation. The adaptive learning and user-customized controlled toxicity vocabularies were applied to extract categorized treatment and prognosis data, so as to generate structured output.

**Results:**

In total, we extracted data from 261 treatment and prognosis documents relating to 50 patients, with overall precision and recall more than 93% and 83%, respectively. For toxicity information extractions, which are important to study patient posttreatment side effects and quality of life, the precision and recall achieved 95.7% and 94.5% respectively.

**Conclusions:**

The IDEAL-X system is capable of extracting study data regarding NSCLC chemoradiation patients with significant accuracy and effectiveness, and therefore can be used in large-scale radiotherapy clinical data studies.

## Introduction

Locally advanced or inoperable non–small cell lung cancer (NSCLC) occurs in approximately 20% to 30% of all cases of NSCLC [1] and may be treated with a combination of definitive concurrent chemotherapy and radiation. Modern radiotherapy has made great advances in the care of NSCLC patients, by reducing potential toxicities using involved field irradiation, while improving survival rates [2-4]. Assessing the effects of new developments in treatment techniques and regimens requires studies on the correlation between the treatment and prognosis [5-7]. Such studies involve extracting extensive patient information on chemoradiation treatments and follow-up assessments, including survival, tumor control, and toxicities.

Information about treatment and prognosis is embedded in treatment summaries and clinical encounter notes, which have various formats and diverse vocabularies. Manual extraction from large volumes of patient treatment summaries and records describing prognosis is time consuming and labor intensive. There is a need for an automated information system, as a natural language processing tool, to extract the needed patient treatment and prognosis data. During recent years, automated information systems have become widely used in medical and biomedical domains. The clinical Text Analysis and Knowledge Extraction System specializes in clinical information extraction [8]. The Cancer Tissue Information Extraction System focuses on annotating cancer text [9]. MedLEE supports connecting value to controlled vocabularies [10]. MedEx aims to extract medication-related information such as dosage and duration [11]. The Clinical Language Annotation, Modeling, and Processing toolkit integrates award-winning algorithms and, moreover, enables users to customize natural language processing components so as to encode clinical text automatically [12,13]. Medical text extraction processes pathology reports and uses rule-based methods to classify lung cancer stages [14]. A recent study also demonstrated that the metastatic site and status of lung cancer could be extracted from pathology reports using a pipeline [15]. Another study showed that cancer stage information could also be extracted with natural language processing [16]. Most traditional information extraction systems rely on batch training or predefined rules and were designed for only limited medical domains or tasks.

**Figure 1:**
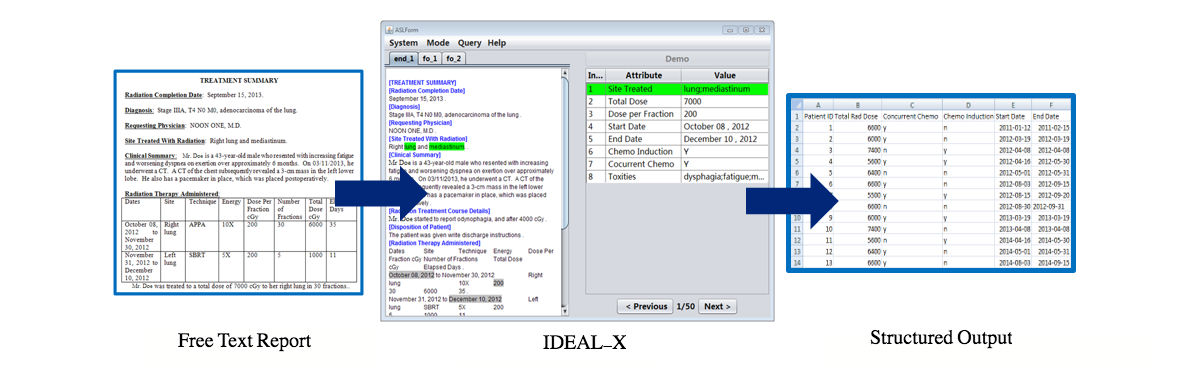
Screenshot of the Information and Data Extraction using Adaptive Learning (IDEAL-X) platform, and example input and output.

To support a retrospective study of NSCLC chemoradiotherapy patients, we adapted our in-house–developed information extraction platform, Information and Data Extraction using Adaptive Learning (IDEAL-X; X represents controlled vocabulary) system [17-19]. This information extraction system aims to transform free-text clinical documents into structured data and has been used by projects in cardiology and pathology. IDEAL-X possesses unique features different from the systems mentioned above: (1) users may freely customize attributes to be extracted; (2) the system extracts information from narrative medical documents and generates normalized values to populate output tables and assist manual annotation; (3) it requires no mandatory configurations or training before performing annotation and adaptive learning processes; and (4) the system learns from users’ normal interactions transparently, and establishes and refines decision models incrementally, which further alleviates manual annotation efforts. Figure 1 shows how the IDEAL-X system processes the input from free-text reports generated during physician and patient encounters and delivers structured output.

## Methods

### Patient Information

We collected NSCLC patient data to investigate the relationship between shrinkage of the treated tumor and each category of prognosis data: survival, tumor control, and toxicities. The patient treatment data we needed to identify included the chemoradiotherapy drugs used, dose, and treatment time frame. From the follow-up clinical notes, we needed to extract tumor control information diagnosed from the patient’s follow-up computed tomography and positron emission tomography images, patient toxicities, and complication data, including skin, internal organ, blood, and overall body reactions to treatment. We further categorized toxicities into different toxicity grades [20]. After we extracted the information in a structured format, we intended to use it to statistically correlate treatment tumor shrinkage with survival time, disease control rate, and the toxicities.

From studies approved by the institutional review boards of both Rutgers University and Emory University, we retrospectively identified 50 patients who had primary unresectable, locally advanced, biopsy-proven stage II-III NSCLC, and who had received chemoradiotherapy with a median follow-up of 22 months. In total, we exported 261 treatment and patient follow-up documents from the patient electronic health record system ARIA (Varian Medical Systems, Inc, Palo Alto, CA, USA) and anonymized the data for this study.

### IDEAL-X System Development

We adapted the IDEAL-X system to support automated information extraction from the NSCLC chemoradiation patients’ documents. After a requirement analysis, we added new features, such as extracting timex and parsing tabular information, to enhance the original system. We also implemented corresponding feature extraction and machine learning processes for timex and tabular formats, and constructed the dictionary to assist toxicity data extraction. We extracted patient information, such as treatment time frame and chemoradiotherapy, from treatment records with an adaptive learning process (Table 1). In extracting this information, the system began without any prior training and created its machine learning model incrementally. During the information extraction of the toxicities, the adaptive learning process was disabled. We used the dictionary shown in Textbox 1 to aid in toxicities information extraction. Along with extracted values, the sentences where the values were embedded were also output in a spreadsheet, which could be used for further manual toxicity grade differentiation based on patient Common Terminology Criteria for Adverse Events guidelines v 4.0, which were designated previously in the patient charts [20].

In addition, to verify the extracted data, we asked 2 physicians to manually annotate these reports. We used the manually annotated ground truth to validate the automatically generated output from the IDEAL-X system. We used precision and recall results to estimate the effectiveness of extraction.

### IDEAL-X Adaptive Learning Process

Through adaptive learning, IDEAL-X established its decision model through ordinary operations in manual annotation. First, the user designated the value to fill every attribute in the structured output form. After a few initial documents, the system quickly learned important and related information that the user sought and began to generate standardized values automatically in subsequent documents. The system continued to learn and update its knowledge, without special user intervention. This incremental learning process made the system domain agnostic and not limited to a specific medical report. When available, a user-defined controlled dictionary and other configurations could also be provided by the user to facilitate this learning process, but they were not mandatory.

### System Data Flow

**Figure 2:**
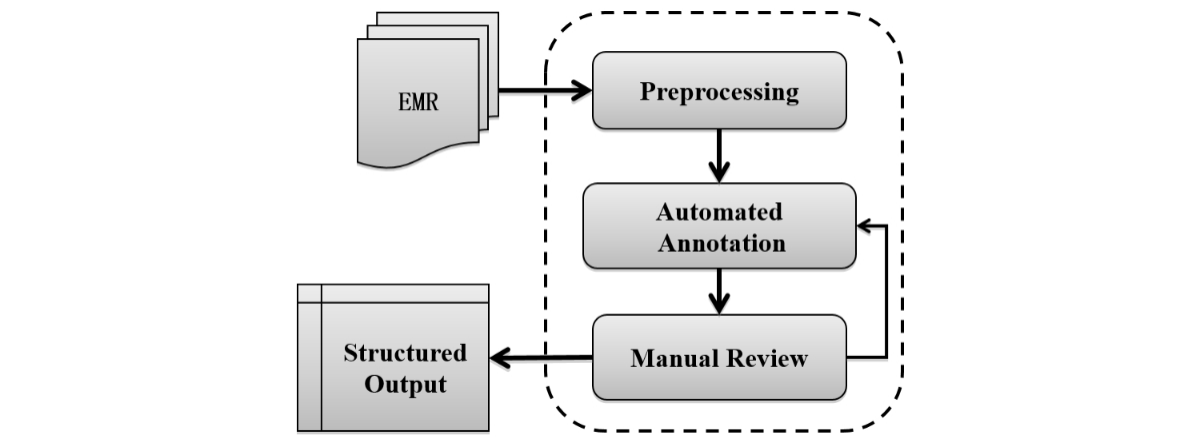
Data flow in the Information and Data Extraction using Adaptive Learning (IDEAL-X) platform. EMR: electronic medical record.

Figure 2 demonstrates the system’s data flow. Each time that the system loaded a document, the system moved through the preprocessing phase and parsed the text to analyze and identify important linguistic features and natural language elements. These features and elements included (1) part of speech: the part-of-speech tag of each word, for example, noun and verb; (2): timex: the system relied on predefined regular expressions to identify timex, such as 2010-01-09 and Sep 13, 2013, and then indexed them based on their position in the text; (3) tabular information: the system identified and parsed tables in input text to comprehend underlying relations between values and the metadata in a table; (4) negation terms: the system detected negation terms and regions being affected, for example, in the case of “patient denies fever and fatigue,” “fever” and “fatigue” were not extracted as part of the toxicities; and (5) uncertain terms: the system identified uncertain phrases and regions being governed, for example, “We explained to her that the risks of the treatment included dysphagia and pneumonitis” meant that dysphagia and pneumonitis had not appeared yet as symptoms. We used these features to mark the input text and provide detailed linguistic indications during extraction.

After preprocessing, the parsed text was investigated by the automated annotation component of the system to populate the output form automatically. First, sentences where possible values may be located were extracted based on text hierarchy, frequently co-occurring terms, previously extracted values, or user-customized vocabularies. The system then identified candidate phrases from located sentences using either a hidden Markov model [21] chunker or a dictionary chunker. Subsequently, candidate values were examined by various filters based on linguistic features such as part of speech, certainty, or negation collected during preprocessing. After filtering, the sentence score and the chunk score were combined, on the basis of which a classifier determined the overall confidence score of each candidate value and categorized it as “accept” or “reject.”

We then reviewed the automatically extracted values manually for the purpose of adaptive learning. We considered positive and negative scenarios: if the user navigated to the next document without changing any values, we regarded the values generated by the system as positive training cases; if the user modified any values, we regarded the system-generated values as negative training cases and the manually updated values as positive ones. We used the results of the review to support further improvements in the automated annotation component. Difference feature extract procedures, which model the traits of numerical, nominal, timex, and tabular data elements, were applied to corresponding positive and negative instances. By repeating these steps, the system became intelligent incrementally and delivered more accurate results.

**Table 1:** Information extracted from treatment records of patients with non–small cell lung cancer.

Attributes	Text data type	Numbers of values	Dictionary	Adaptive learning
Treatment site	Nominal	68	N/A^a^	Yes
Chemotherapy information	Nominal	56	N/A	Yes
Treatment time frame	Date	92	N/A	Yes
Radiation therapy dose	Numerical	97	N/A	Yes
Toxicities	Nominal	331	Yes	N/A

^a^N/A: not applicable.

**Dictionary of toxicities.**

Anemia

Lymphopenia

Anorexia

Dehydration

Dyspnea

Fatigue

Mucosal inflammation

Radiation esophagitis

Weight decrease

Cough

Febrile neutropenia

Neutropenia

Bronchitis

Diarrhea

Esophagitis

Hyponatremia

Nausea

Radiation pneumonitis

Dermatitis

Leukopenia

Thrombocytopenia

Decreased appetite

Dysphagia

Failure to thrive

Localized infection

Pneumonia

Vomiting

Insomnia

## Results

**Figure 3:**
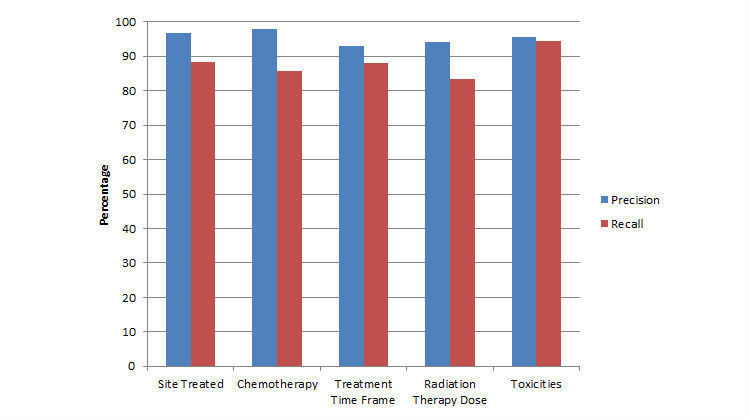
Effectiveness of data extraction as estimated by precision and recall of automatically generated output compared with manually annotated ground truth.

Figure 3 shows the validation results against the manually annotated ground truth. In the validation for patient characteristics and tumor control, the system achieved an overall precision of over 93%. The recall values of all information were more than 83%. The recalls were lower than the precisions, as the recalls reflected the performance during the overall adaptive learning process—the system processed a few documents to construct and refine its decision model at its early stage in the adaptive learning process.

Especially in the extraction of the toxicities, the negation detection and certainty detection filters contributed directly to the accuracy of extraction. With the help of a controlled dictionary, the system achieved an overall precision of 95.7% and recall of 94.5%.

Within 1 second, a well-trained system can process patient documents of multiple pages and output the results in a predefined format. Compared with manual review, which requires reading through the entire document and manually annotating the notes on each patient, this system significantly improved the efficiency of information extraction.

## Discussion

IDEAL-X employed adaptive learning and a controlled vocabulary to support information extraction, which alleviated both the training and the deployment processes that could be expensive in applying a traditional information extraction system. The various data types IDEAL-X supports cover the most important and common information in oncology reports, which delivers great usability to our use case. We have demonstrated the great advantage of this system in greatly improving information extraction effectiveness while maintaining high accuracy when applied to extracting NSCLC patient treatment and prognoses data from heterogeneous document formats. In addition, because the system improves its performance incrementally, its accuracy could be further improved with additional training documents. Once trained, the developed system was able to process further fed-in reports in batch mode without revision. Without an intervening regular manual reporting process that handles input documents in sequence, the system accumulates knowledge transparently to empower the task and, therefore, could be conveniently integrated into a regular clinical workflow. The technology it used was domain agnostic and, therefore, could be transformed to other disease sites and studies in radiation oncology.

### Limitations

In the validation analysis, the system also revealed some unavoidable limitations. The system identified and comprehended information based on explicitly expressed keywords. For example, the phrases “neoadjuvant chemo” and “upfront chemotherapy” may be used as keywords to identify chemotherapy induction. However, in situations where relevant information is distributed across different regions in the text, more insightful comprehension becomes necessary. For example, in the case of “After 4 cycles of chemotherapy and abdomen...we began radiation...,” the system was not intelligent enough to interpret the meaning of “4 cycles” as “neoadjuvant chemotherapy” behind the narrations. In general, this sophisticated scenario reveals the limitation of this information extraction-based approach. The system requires explicit keywords or hints to determine an event; however, it cannot reason and analyze factors collected from different sources. Such cases resulted in lower recalls for chemotherapy than for other attributes and demanded a manual review. Therefore, to facilitate the manual review, we output the associated sentence with the extracted information together in tabular format for user manual review and validation at a later time.

### Conclusion

We adapted the IDEAL-X system to automatically extract treatment and prognostic information for stage II and III NSCLC patients who had received chemoradiation. With this system, patient information was extracted efficiently from their medical documents in various formats. The system, together with minimized manual review efforts, generated outputs with high precision and recall. It significantly improved the effectiveness and can be easily applied to other radiation oncology patient studies at larger scales.

## Abbreviations

EMR: electronic medical record
IDEAL-X: Information and Data Extraction using Adaptive Learning
NSCLC: non–small cell lung cancer
